# Exposure to p,p’-dichlorodiphenyldichloroethylene (p,p’-DDE) Perturbs Cell State and Stem Cell Markers and Induces an Epidermal Phenotype in Human Breast Cells

**DOI:** 10.64898/2026.10.13.758898

**Published:** 2026-10-20

**Authors:** Jenna L. Miller, Anagha Tapaswi, Jesse W. Wotring, Katelyn M. Polemi, Penghui Yang, Xiang Zhou, Laurie K. Svoboda, Jonathan Z. Sexton, Justin A. Colacino

## Abstract

Breast cancer is the second leading cause of cancer deaths in U.S. women. Endocrine disruptors, such as the DDT metabolite DDE, are environmental factors linked to breast cancer risk. Emerging evidence shows that during carcinogenesis, cells reacquire stem cell traits and exhibit phenotypic plasticity. In these phenotypically plastic states, breast cells transition between phenotypes, for example, from epithelial to mesenchymal (EMT) phenotypes, or from luminal to basal. Discovering how environmental factors alter cell phenotype may be a critical step in preventing and treating breast cancer. We applied a novel high-content imaging method for the evaluation of expression of stemness and cell state markers (ALDH1A3, CD44, CD24, KRT8, and KRT14) and evaluated these markers after introducing environmentally relevant concentrations of DDE (25nM to 25µM) to non-tumorigenic MCF10A breast epithelial cells grown in 3D anchorage independent conditions. After identifying surprisingly profound cell state alterations, including a loss of KRT8 and KRT14 expression, by 25 µM DDE exposure, we used transcriptomic profiling along with pharmaceutical and genetic knockdown approaches to characterize the molecular mechanisms by which DDE exposure modulates breast epithelial cell state. Keratinization and formation of the cornified envelope were biological pathways upregulated with a maximum fold change of 360.47. We identify *DLX3* as a transcription factor which is inducible by DDE exposure and is also highly upregulated in human breast cancers, reflecting a novel mechanism by which environmental exposures may promote cellular plasticity and breast carcinogenesis.

